# Mendelian Randomization Studies on Nutritional Factors and Health Outcomes

**DOI:** 10.3390/nu14142780

**Published:** 2022-07-06

**Authors:** Susanna C. Larsson

**Affiliations:** 1 Unit of Cardiovascular and Nutritional Epidemiology, Institute of Environmental Medicine, Karolinska Institutet, 17177 Stockholm, Sweden; susanna.larsson@ki.se; 2 Unit of Medical Epidemiology, Department of Surgical Sciences, Uppsala University, 75185 Uppsala, Sweden

Poor diet is a leading cause of morbidity and mortality [1,2]. However, the causal roles of specific foods, nutrients, and other dietary factors in health and disease are not fully established, as most evidence originates from conventional observational studies. In most of those studies, information on diet and other potential risk factors for diseases was obtained through questionaries or interviews, and the data were sometimes complemented with measured biomarkers. Such studies are susceptible to misclassification of dietary intake as well as residual confounding from correlated factors and reverse causation bias. Mendelian randomization (MR) is a method that can reduce such biases in observational studies and provide more robust evidence concerning the role of dietary factors in health and disease.

Studies published in this Special Issue applied the MR design to investigate the potential causal associations of higher exposure to milk, alcohol, coffee, caffeine, and different nutrients with risks for various diseases. Zhang et al. examined the associations of genetically predicted milk, alcohol, and coffee consumption with risk of neurological and neurodegenerative diseases [3,4]. Milk consumption was proxied by a genetic variant near the *LCT* gene, encoding the lactase enzyme which digests milk sugar. The used genetic variant is associated with milk consumption in European populations. Findings from the MR studies by Zhang et al. provided evidence that milk consumption may reduce the risk of epilepsy [4], multiple sclerosis, and Alzheimer’s disease but may increase the risk of Parkinson’s disease [3]. Genetically predicted alcohol consumption was associated with an increased risk of epilepsy, whereas no association was observed between genetically predicted coffee consumption and epilepsy risk [4]. Another MR study published in this Special Issue investigated the associations of higher plasma caffeine levels instrumented by genetic variants involved in caffeine metabolism with the risk of Alzheimer’s disease and Parkinson’s disease [5]. That study found possible suggestive evidence of a protective role of caffeine in Alzheimer’s disease [5].

Three MR studies evaluated the associations of genetically predicted levels of circulating nutrients, including fatty acids, amino acids, vitamins, and minerals, with risk of amyotrophic lateral sclerosis [6], chronic kidney disease [7], and COVID-19 [8]. Xia et al. found that genetically predicted linoleic acid was positively associated with amyotrophic lateral sclerosis, whereas genetically predicted vitamin D and vitamin E levels were inversely associated with this disease [6]. No significant association was observed between genetically predicted circulating levels of essential amino acids and minerals and risk of amyotrophic lateral sclerosis [6]. The MR study by Ahmad et al. found evidence that elevated circulating copper levels may be a causal risk factor for chronic kidney disease [7]. Sobczyk and Gaunt assessed whether genetically predicted higher circulating levels of copper, zinc, selenium, or vitamin K_1_ within the usual range have a causal association with COVID-19-related outcomes, including risk of infection, hospitalization, and critical illness [8]. They observed no significant associations but acknowledged several limitations [8]. There were concerns about the validity of the results for vitamin K_1_, and there were concerns that vitamin K_2_, for which there are no available genetic instruments, may be more important particularly during acute illness [8,9,10].

Taken together, these MR studies have added to the evidence that milk consumption may have a role in neurogenerative diseases and that elevated caffeine levels might reduce the risk of Alzheimer’s disease. The possible role of nutrients in amyotrophic lateral sclerosis, chronic kidney disease, COVID-19, and other diseases needs further study. I hope that readers of this Special Issue will find inspiration for further MR analyses to decipher the role of dietary factors in chronic diseases.
